# Use of Nomogram to Predict the Risk of Lymph Node Metastasis among Patients with Cervical Adenocarcinoma

**DOI:** 10.1155/2022/6816456

**Published:** 2022-08-23

**Authors:** Yongju Tian, Yiping Hao, Qingqing Liu, Ruowen Li, Zhonghao Mao, Nan Jiang, Bingyu Wang, Wenjing Zhang, Xiaofang Zhang, Baoxia Cui

**Affiliations:** ^1^Department of Obstetrics and Gynecology, Qilu Hospital of Shandong University, Jinan, Shandong, China; ^2^Department of Gynecology, Yantaishan Hospital, Yantai, Shandong, China; ^3^Department of Pathology, School of Basic Medical Science, Shandong University, Jinan, Shandong, China; ^4^Department of Pathology, Qilu Hospital of Shandong University, Jinan, Shandong, China

## Abstract

**Background:**

The objective of this study was to develop a nomogram that can predict lymph node metastasis (LNM) in patients with cervical adenocarcinoma (cervical AC).

**Methods:**

A total of 219 patients with cervical AC who had undergone radical hysterectomy and lymphadenopathy between 2005 and 2021 were selected for this study. Both univariate and multivariate logistic regression analyses were performed to analyze the selected key clinicopathologic features and develop a nomogram and underwent internal validation to predict the probability of LNM.

**Results:**

Lymphovascular invasion (LVI), tumor size ≥ 4 cm, and depth of cervical stromal infiltration were independent predictors of LNM in cervical AC. However, the Silva pattern was not found to be a significant predictor in the multivariate model. The Silva pattern was still included in the model based on the improved predictive performance of the model observed in the previous studies. The concordance index (*C*-index) of the model increased from 0.786 to 0.794 after the inclusion of the Silva pattern. The Silva pattern was found to be the strongest predictor of LNM among all the pathological factors investigated, with an OR of 4.37 in the nomogram model. The nomogram developed by incorporation of these four predictors performed well in terms of discrimination and calibration capabilities (*C* − index = 0.794; 95% confidence interval (CI), 0.727–0.862; Brier score = 0.127). Decision curve analysis demonstrated that the nomogram was clinically effective in the prediction of LNM.

**Conclusion:**

In this study, a nomogram was developed based on the pathologic features, which helped to screen individuals with a higher risk of occult LNM. As a result, this tool may be specifically useful in the management of individuals with cervical AC and help gynecologists to guide clinical individualized treatment plan.

## 1. Introduction

Cervical cancer is among the most prevalent cancer among women, with approximately 604,000 new cases and 342,000 mortalities across the globe in 2020 [1, 2]. Cervical adenocarcinoma (cervical AC) is the second most common histological type of cervical cancer and accounts for 20–25% of all cervical malignancies. The real and relative evidence suggests an increasing trend in this type of cancer over the past 20 years [3]. Additionally, cervical AC cases are rising in the younger population and an increasing number of cervical AC patients particularly belong to the reproductive age [3]. Similar to most other cancers, the preferred treatment for cervical cancer at an early stage is radical surgery. Surgery can remove the primary lesion and facilitate the selection of postoperative adjuvant therapy by precise surgical staging [4]. Lymph node metastasis (LNM) is a significant prognostic factor in early cervical cancer and has a poor prognosis; therefore, patients with LNM should undergo postoperative treatment, which means that evaluating the status of lymph nodes is critical [5].

Lymphadenectomy is widely used to evaluate the status of lymph nodes in early cervical malignancy; however, there are still controversies regarding its application principle [6]. Many people who underwent lymphadenectomy are found to be suffering from lymphadenectomy-related morbidities, including lymphedema, urinary dysfunction, and nerve-site injury, which seriously affect the quality of life of the patients [8, 9]. However, only a small number of patients in the early stage have LNM [7]. But a question that arises here is whether cervical cancer patients are undergoing excessively lengthy treatment. There is some evidence that sentinel lymph node (SLN) biopsy might serve as a lymph node staging method in individuals with early cervical malignancy. However, this technique has not yet been widely validated [10–14]. Therefore, the identification of patients with a low risk of LNM before undergoing lymphadenectomy is crucial.

Late-stage, lymphovascular invasion (LVI) and destructive stromal invasion are recognized as poor prognostic factors of cervical AC [15]. In 2013, Diaz et al. suggested a new system for usual-type cervical AC based on the stromal invasion pattern, which categorizes cervical AC patterns into three types: pattern A (well-demarcated glands), pattern B (early destructive stromal invasion arising from well-demarcated glands), and pattern C (diffuse destructive invasion). This new stratification system indicates that patients with pattern A tumors can be spared lymphadenectomy, patients with pattern B tumors can be potentially identified by SLN examination, and lymphadenectomy is justified in patients with pattern C tumors [16, 17]. Therefore, this new system is successful in considerably reducing the frequency of needless radical lymphadenectomy among early-stage patients. Previous studies have highlighted that when compared with the clinical stage, this new system helped to accurately predict the risk of LNM [15–18].

This study is aimed at developing a nomogram that could predict LNM in cervical AC patients and at using the nomogram to identify patients at a low risk for LNM, thus providing information for indicating the need for adjunctive therapy and determining prognosis.

## 2. Materials and Methods

### 2.1. Patients

This study retrospectively included 243 cervical AC patients who underwent surgery at Qilu Hospital of Shandong University and the Second Hospital of Shandong University between April 2005 and March 2021. This research followed the approval documents from the institutional review board. The inclusion criteria were as follows: pathologically confirmed cervical adenocarcinoma, a clinical diagnosis of International Federation of Gynecology and Obstetrics (FIGO) stage IA-IIA disease, radical hysterectomy with pelvic and/or para-aortic lymphadenectomy, and no history of chemotherapy or radiotherapy. We excluded patients with other types of cervical cancer, including squamous cell carcinoma, adenosquamous carcinoma, and other special types which could not be Silva classified. Patients who did not undergo lymphadenectomy were also excluded. A total of 219 patients satisfied the eligibility criteria.

### 2.2. Preoperative Assessment

To identify variables predicting LNM, the following factors were included: age, FIGO stage, tumor size, and variables of pathologic characteristics. The stage of cervical AC was based on the standard FIGO criteria [19]. Diagnosis of stage IA1 and IA2 was based on endoscopic examination of the intact lesion by loop electrosurgical excision procedure (LEEP) or cold knife conization (CKC) and could also be made with cervical or total hysterectomy specimens. Biopsy was used to diagnose the lesion visible to the gross eye. In unsatisfactory cases, small LEEP or CKC was used. For primary tumors > 10 mm in diameter, tumor size was assessed by magnetic resonance imaging (MRI). The results of preoperative biopsy, LEEP or CKC, pelvic MRI, and whole-body positron emission tomography/computed tomography (PET/CT) were interpreted by pathologists, radiologists, and nuclear medicine physicians, respectively.

### 2.3. Pathology Data Abstraction

Pathologic data were retrieved from the pathology reports which included information on tumor grade, tumor size, LVI, myometrium invasion, vaginal and parametrium involvement, depth of cervical stromal infiltration, number of lymph nodes selected and involved, and Silva pattern. The Silva pattern was based on the morphological characteristics of the invasive carcinoma. In compliance with the criteria for the Silva pattern system, two senior pathologists assessed all the slides and classified patients into patterns A, B, or C by unanimity (Supplementary Table 1). In this study, there was no LNM in patients with the Silva pattern A, only 1 out of 27 patients with the Silva pattern B had LNM, and 41 out of 171 patients with the Silva pattern C had LNM. Therefore, based on the LNM results, patients with the Silva pattern A and pattern B were classified as low-risk groups and patients with pattern C as a high-risk group. The largest measurement of horizontal spread or surface diameter in the field was used to define tumor size in this investigation. Based on the size, the tumors were categorized into two groups: <4 cm and ≥4 cm.

### 2.4. Statistical Analysis

Both the univariate and multivariate logistic regression analyses were performed to evaluate the relationship between the pathologic characteristics and the incidence of LNM. Pathologic factors that were not significant or regarded as not clinically relevant were removed to construct an association model with excellent predictive power. Although no significant differences in the Silva system were observed during multivariate regression, the Silva pattern was included as a variable in this study because several studies have established the accuracy of the Silva pattern to predict LNM [15–18]. Besides, the predictive performance of the developed model was improved after the inclusion of the Silva pattern in the final model.

A nomogram was developed based on the final multivariate regression model to predict LNM. The internal validation of the nomogram was conducted by the jackknife cross-validation test. Further, the risk of LNM was computed for every patient based on the nomogram fitted to the remaining data, and the ability of the model to distinguish between the individuals was measured by the concordance index (*C*-index). The developed model was characterized based on the area under the curve (AUC) and receiver operating characteristics. All the statistical tests were two-sided, with *p* values < 0.05, indicating statistical significance. The statistical analyses were conducted using Stata/MP 16 and R software (https://www.r-project.org/, version 4.0.5). The rms package in R was used to plot the nomogram, pROC package was used to plot the ROC curve, and rmda package was used to plot the decision curve analysis (DCA).

## 3. Results

### 3.1. Patient and Pathologic Characteristics

In this study, 219 patients were recruited based on the inclusion criteria of the research. At the time of staging, the participants had a median age of 46.5 (range, 22–69 years). The average total of removed lymph nodes was 20.7 (range, 6–52). Among all the patients, 42 (i.e., 19.18%) had at least one LNM.  Table 1 lists the detailed pathologic findings at the time of surgical staging.

### 3.2. Univariate and Multivariate Predictors for Lymph Node Metastasis

The univariate analysis indicated that tumor size ≥ 4 cm, LVI, parametrial involvement, depth of cervical stromal infiltration, FIGO stage, and Silva pattern were notably linked to LNM. However, factors such as vaginal involvement, age, grade, profession, marital status, smoke, and whether the patients underwent laparoscopic surgery were not linked to LNM. In a multivariate model, LVI, tumor size ≥ 4 cm, and depth of cervical stromal infiltration were found to be viable predictors of LNM. The findings of the univariate and multivariate logistic regression analysis are summarized in Table 2.

As LVI, tumor size ≥ 4 cm, and depth of cervical stromal infiltration were observed to be the significant predictors of LNM in multivariate regression analysis, these factors were included in the developed model. Moreover, as several studies have shown that the Silva pattern can accurately predict LNM, it was also incorporated into the model [15–18]. Before the addition of the Silva pattern into the model, the *C*-index of the model was 0.786 and increased to 0.794 after the inclusion of the Silva pattern. Thus, it can be observed that the Silva pattern can improve the prediction accuracy of the model (Supplementary Figure 1).

### 3.3. Nomogram for Predicting Risk of Lymph Node Metastasis

**Figure 1:**
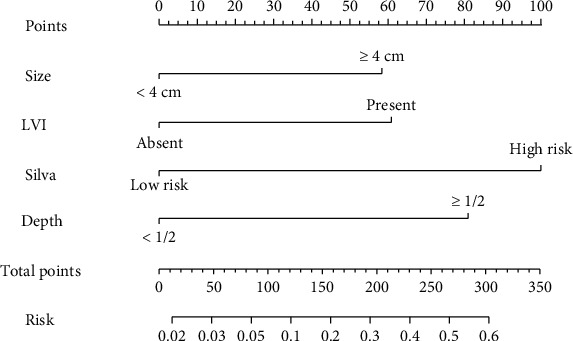
Nomogram for predicting LNM.

A nomogram was constructed based on the final multivariate model and the clinical practices to quantify the risk of LNM (Figure 1). Tumor size ≥ 4 cm, depth of cervical stromal infiltration, LVI, and Silva pattern were included in the nomogram as the predictor variables, and the weighted points were allocated to each variable for individualizing risk prediction. An anticipated probability of LNM is equal to the sum of all points. A vertical line is drawn for each variable point on the axis to compute the probability of LNM. The scores of all the variables were calculated and summed to obtain the total score. A vertical line is projected from the total score line to the predicted probability bottom scale to obtain the individual probability of LNM. Among all the pathologic characteristics investigated, the Silva pattern was shown to be the strongest predictor of LNM, with an odds ratio (OR) of 4.37 in the model. LVI was found to be a viable biomarker for LNM. The specific scores of the variables in the developed model are shown in Supplementary Table 2. The risk stratification corresponding to the total scores is shown in Supplementary Table 3.

In clinical practice, the nomogram can be utilized to anticipate the cervical AC among patients. For example, an individual with a primary tumor size of 45 mm (58.5 points), LVI (60.9 points), Silva C (100 points), and depth of cervical stromal infiltration > 0.5 (81.0 points) had a total score of 300.4 points. The corresponding LNM probabilities were found to be more than 60%.

**Figure 2:**
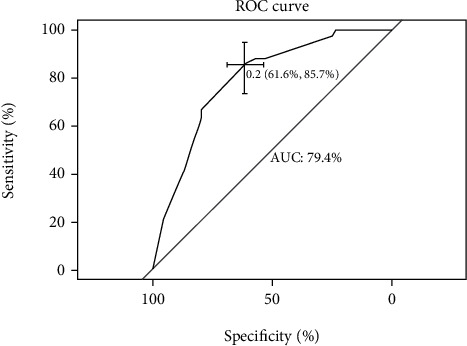
ROC curve of the developed model.

**Figure 3:**
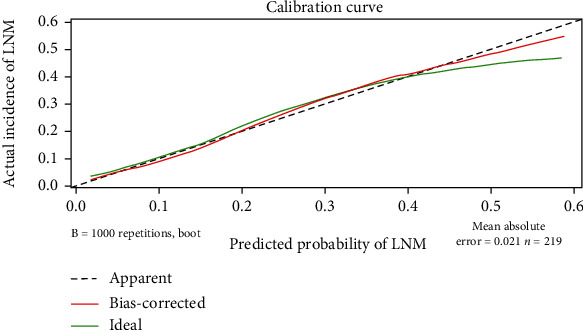
Internal verification plots of the nomogram calibration curves by 10-fold cross-validation.

**Figure 4:**
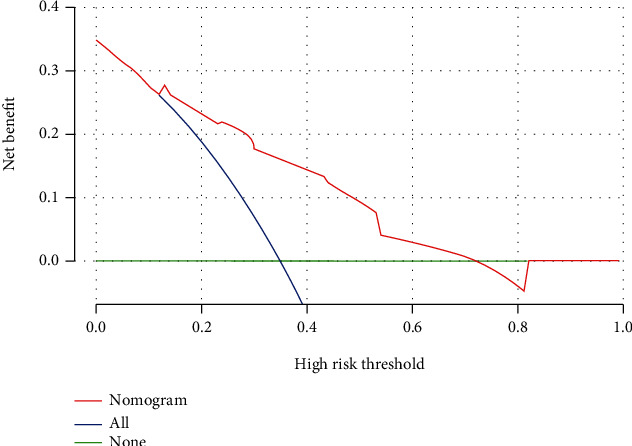
Decision curve analysis of the nomogram for predicting LNM.

The internal cross-validation of the nomogram yielded an approximate *C*-index of 0.794 (*C*-index values of 1.0 and 0.5 indicate a perfect *C*-index and no relationship, respectively). The *C*-index was almost equal to the AUC of receiver operating characteristic curve (ROC), which plotted the sensitivity (true positive rate) against 1 − specificity (false positive rate) to predict the outcome (Figure 2). The AUC for the nomogram constructed to predict LNM was 0.794 (95% CI, 0.727 to 0.862). The Brier score was used to check the overall performance of the model [20]. In the internal validation, the optimism-corrected Brier score for the prediction of LNM was 0.127 (scores of 0 and 1 indicate perfect prediction accuracy and poor prediction accuracy, respectively).  Figure 3 depicts how the predictions from the nomogram compare with the actual outcomes for the 219 patients in this study. DCA was applied to evaluate the clinical benefit of the nomogram. As shown in Figure 4, the nomogram demonstrated good predictive ability across wide ranges of LNM risk, indicating its effective clinical utility in the prediction of LNM.

## 4. Discussion

Lymphatic metastasis is the most common phenomenon in cervical AC, as well as a vital prognostic factor affecting recurrence and survival. However, routine lymphadenectomy has been a controversial issue [21, 22]. In recent years, as many young patients are diagnosed with cervical cancer, younger age at onset is observed [3]. Lymphadenectomy not only increases operating time and blood loss but also leads to many severe complications, such as chronic lymphedema, lymphocysts, infection, and vessel and nerve injuries, thus seriously impacting the quality of life in patients with cervical AC [8, 9]. With a younger age of onset of cervical AC, it is necessary to recruit the patients who significantly benefit after lymphadenectomy in addition to identifying noninvasive patients and reducing harmful lymph node assessment. To achieve this goal, a nomogram was developed based on the pathologic factors to determine the risk of LNM.

A few researchers have made some progress toward accurately determining the risk of LNM using preoperative imaging data such as PET/CT and MRI combined with clinical data to predict LNM [23–25]. Several meta-analyses were conducted to evaluate the diagnostic performance of CT, MRI, and PET in patients with cervical cancer [24, 25]. However, the capacity of the existing imaging methods to determine microscopic nodal diseases is limited [23]. Therefore, many scientists have also proposed the use of SLN biopsy to evaluate the status of lymph nodes. This procedure requires an injection of a dye into the cervix, which further spreads to the sentinel nodes. However, its routine clinical application is still limited because of technical difficulties such as intraoperative frozen sections and ultrastaging. Moreover, Cibula et al. emphasized that this technique is unfeasible in clinical practice because the identified micrometastases (MIC) and isolated tumor cells (ITCs) need hundreds of slides per SLN. Therefore, it is evident that a certain proportion of MIC and ITCs is represented in SLN-negative patients [10]. More studies are necessary to confirm the feasibility of SLN biopsy in cervical malignancy [11, 13].

Recently, researchers have become increasingly interested in nomogram. It is an intuitive and easily readable graphical chart based on the logistic regression or Cox regression and could accurately predict the probability of occurrence of an event. For malignant tumors, the most important benefit of nomogram is that the risk can be assessed by noninvasive or minimally invasive procedures before radical surgery. Individualized prediction based on the nomogram could help inform decision-making by surgeons and patients. Kim et al. developed a robust model incorporating preoperative variables to predict LNM in patients with early cervical cancer. The model accurately identifies patients at low risk of LNM [26]. Other studies have also shown a nomogram was developed to improve the ability to predict the risk of LNM, such as gallbladder cancer, endometrial cancer, and gastric cancer [27–29]. The nomograms based on clinicopathological parameters have good ability to predict the risk of LNM and make up for the inaccuracy of imaging.

In this study, tumor size ≥ 4 cm, LVI, parametrial involvement, depth of cervical stromal infiltration, stage, and Silva pattern were found to be significantly linked to LNM. In a multivariate model, LVI, tumor size ≥ 4 cm, and depth of cervical stromal infiltration were observed to be the viable predictors of LNM, while the Silva pattern was not found to be a viable predictor. However, a study by Diaz et al. compared pattern A with pattern C, and the difference in LNM was found to be notably significant [16]. Besides, Byun et al. evaluated 76 cases of invasive cervical AC and observed that among those with usual and variants, all patients with pattern A tumor had no LNM and did not develop recurrence. A study conducted by Byun et al. among 76 study subjects ascertained that each patient with pattern A tumor did not experience LNM and recurrences among patients with usual and the variants [30]. The model developed in this study includes the Silva pattern, which led to the improved accuracy of the nomogram model [15–18]. However, a drawback of the developed model may be the small sample size considered in this study.

In this study, no LNM was identified in patients with the Silva pattern A, only one of 27 patients with the Silva pattern B had LNM, and 41 out of 171 patients with the Silva pattern C had LNM similar to that observed in other studies. Among the 171 patients with the Silva pattern C, 52 patients had tumor diameter < 4 cm, no LVI, and cervical invasion depth < 0.5 and only 4 patients had LNM. Due to a large proportion of cervical AC patients with the Silva pattern C, it is unscientific to treat all pattern patients in the same manner. These findings are similar to those reported in the study by Alvarado-Cabrero et al. [31]. They found six architectural growth patterns of the Silva pattern C and different recurrence rates and prognosis of patients with different growth patterns. That study also demonstrated that all the Silva pattern C patients do not show aggressive behavior. As a result, additional methods and indicators are needed to screen patients at risk of occult nodal disease and safeguard the fertility of young patients. In this study, pathologic variables that indicate a high risk of LNM were identified and a nomogram was developed to evaluate this risk to assist in predictions and adjuvant therapy decisions. The *C*-index of the lymph node predictive nomogram was 0.794. In general, the Silva pattern was found to be the most crucial predictor of LNM. This is the first study to consider the Silva pattern in conjunction with other clinicopathological factors to determine its accuracy in predicting LNM.

This study has some unique advantages:(1) the pathological features of the tumor; namely, Silva classification, LVI, depth of cervical stromal infiltration, and tumor diameter, could be obtained by complete resection of the tumor through loop electrosurgical excision procedure (LEEP) or cold knife conization, and (2) the clinicians can use this model to evaluate the probability of LNM and make a decision on the next line of treatment based on the results. According to the developed model, hysterectomy or cervical conization with a negative margin is feasible in patients with a probability of LNM < 5% [26]. When the model predicts a low rate of LMN in patients with the Silva pattern C, radical hysterectomy and SLN biopsy may be more feasible treatment options. The model can also be used to evaluate the probability of LNM and to guide postoperative adjuvant treatment in patients who have not undergone lymph node surgery. A high probability of LNM indicates the need for further radiotherapy and chemotherapy, while a very low probability of LNM indicates regular follow-up.

However, the developed model also has some limitations. Despite the model being internally validated, there is still a need for independent samples for external validation to ensure the stability of the model. The clinical and pathological characteristics of samples from different medical institutions may lead to potential heterogeneity. There is still no uniform standard for adequate lymphadenectomy, and this study included patients with any of the lymph nodes removed. According to the European Organization for Research in the Treatment of Cancer-Gynecological Cancer Group (EORTC-GCG), the minimum number of removed pelvic lymph nodes is 12 [32]. Besides, the International Union against Cancer proposed that at least 10 lymph nodes should be investigated for the determination of pN0 [33]. Thus, it is not clear whether a minimum number of lymph nodes should be considered in lymphadenectomy and whether the number of lymph nodes removed is linked to prognosis in cervical cancer. In this study, the number of lymph nodes removed from patients ranged from 6 to 52 (median, 20.7).

## 5. Conclusions

In conclusion, a nomogram was developed based on clinicopathological parameters to assist in the identification of individuals at risk of occult LNM. This method can be very beneficial in the treatment of cervical AC patients and may facilitate gynecologists to guide clinical individualized treatment plan. Future studies should focus on expanding the sample size to further verify the feasibility of the model. Moreover, there is a need to carry out randomized controlled studies to further verify the clinical value of the model.

## Figures and Tables

**Table 1:** Variables of pathologic characteristics.

Variable		Total (*n* (%))
Lymph node positive	No	177 (80.82)
	Yes	42 (19.18)
LVI	Absent	173 (79)
	Present	46 (21)
Silva pattern	Pattern A	21 (9.59)
	Pattern B	27 (12.33)
	Pattern C	171 (78.08)
Silva pattern	Low risk	48 (21.92)
	High risk	171 (78.08)
Marital status	Unmarried	2 (0.91)
	Married	208 (94.98)
	Divorced	9 (4.11)
Parametrium involvement	No	207 (94.52)
	Yes	12 (5.48)
Vaginal involvement	No	218 (99.54)
	Yes	1 (0.46)
Grade	High	25 (11.42)
	Moderate	76 (34.70)
	Poor	67 (30.59)
	NOS	51 (23.29)
Smoke	No	215 (98.17)
	Yes	4 (1.83)
Depth of cervical stromal infiltration	<1/2	113 (51.6)
	≥1/2	106 (48.4)
Laparoscopic surgery	No	166 (75.8)
	Yes	53 (24.2)
FIGO stage	IA	10 (4.57)
	IB	191 (87.21)
	IIA	18 (8.22)
Profession	Farmer	71 (32.42)
	Nonfarmer	148 (67.58)
Size	<4 cm	71 (32.42)
	≥4 cm	148 (67.58)
Age (years)	[Median (range)]	46.30594 (22-69)
Size (cm)	[Median (range)]	2.734247 (0.2-7.5)
Gravidity	[Median (range)]	3 (0-8)
Pregnancy	[Median (range)]	1.72093 (0-5)
Number of lymph node resection	[Median (range)]	20.7032 (6-52)
Number of lymph node positive	[Median (range)]	0.5342466 (0-11)

Abbreviations: LVI: lymphovascular invasion; FIGO: International Federation of Gynecology and Obstetrics.

**Table 2:** Univariate and multivariate logistic regression analyses of variables.

Variable		Univariate		Multivariate	
	Character	OR (95% CI)	*p* value	OR (95% CI)	*p* value
LVI	Absent	Reference		Reference	—
	Present	3.99 (1.92-8.30)	0.0003^∗^	2.31 (1.03-5.20)	0.043^∗^
Silva	Low risk	Reference		Reference	—
	High risk	14.82 (1.98-110.80)	0.009^∗^	3.38 (0.40-28.61)	0.264
Marital status	Married	Reference		—	—
	Unmarried	Empty		—	—
	Divorced	Empty		—	—
Parametrium involvement	No	Reference		Reference	—
	Yes	4.75 (1.45-15.57)	0.01^∗^	2.24 (0.61-8.30)	0.226
Vaginal involvement	No	Reference		—	—
	Yes	—		—	—
Grade	High	Reference		—	—
	Moderate	2.83 (0.60-13.34)	0.189	—	—
	Poor	3.61 (0.77-17.0)	0.105	—	—
	NOS	2.46 (0.49-12.38)	0.274	—	—
Smoke	No	Reference		—	—
	Yes	1.41 (0.14-13.95)	0.766	—	—
Depth of cervical stromal infiltration	<1/2	Reference		Reference	—
	≥1/2	7.46 (3.14-17.74)	0.000^∗^	3.01 (1.13-8.04)	0.028^∗^
Laparoscopic surgery	No	Reference		—	—
	Yes	0.57 (0.24-1.37)	0.209	—	—
FIGO stage	IA	Reference		Reference	—
	IB	0.35 (0.1-0.97)	0.044^∗^	0.47 (0.15-1.52)	0.209
	IIA	Omitted		Omitted	
Profession	Farmer	Reference		—	—
	Nonfarmer	0.50 (0.25-1.00)	0.051	—	—
Size	<4 cm	Reference		Reference	—
	≥4 cm	4.80 (2.35-9.80)	0.000^∗^	2.42 (1.08-5.39)	0.031
Age (years)		1.01 (0.98-1.05)	0.538	—	—
Size (cm)		1.65 (1.32-2.07)	0.000^∗^	—	—
Gravidity		1.00 (0.80-1.24)	0.968	—	—
Pregnancy		1.45 (0.96-2.19)	0.077	—	—
Number of lymph node resection		1.45 (0.96-2.19)	0.056	—	—

Abbreviations: LVI: lymphovascular invasion; FIGO: International Federation of Gynecology and Obstetrics.

## Data Availability

The data used to support the findings of this study are included within the article.

## Supplementary Materials

Supplementary Materials Supplementary Table 1: Silva pattern system criteria. Supplementary Table 2: scores of the variables in the model. Supplementary Table 3: risk stratification corresponding to the total score. Supplementary Figure 1: AUC of a model without the Silva pattern.
