# Supplementary material for: Use of Nomogram to Predict the Risk of Lymph Node Metastasis among Patients with Cervical Adenocarcinoma
Source: J Immunol Res. 2022 Aug 23;2022:6816456. doi: 10.1155/2022/6816456 (PMC9427274; doi:10.1155/2022/6816456)
Supplement: Supplementary Materials — Supplementary Table 1: Silva pattern system criteria. Supplementary Table 2: scores of the variables in the model. Supplementary Table 3: risk stratification corresponding to the total score. Supplementary Figure 1: AUC of a model without the Silva pattern. [file 6816456.f1.docx]

**Supplementary Table 1 Silva pattern system criteria**

| The Silva pattern system criteria | |
| --- | --- |
| Pattern A | Well-demarcated glands with rounded contours, frequently forming groups  No single cells or desmoplastic stromal reaction Irrelevant relationship to large cervical vessels or depth of the tumor  Complex intraglandular growth allowed (i.e. cribriform, papillae)  No lymph-vascular invasion Well or moderate differentiation |
| Pattern B | Early destructive stromal invasion arising from well-demarcated glands (Pattern A-like glands) ± Lymph-vascular invasion |
| Pattern C | Diffuse destructive invasion |

**Supplementary Table 2 Scores of the variables in the model**

|  | | OR | Coefficient | Points |
| --- | --- | --- | --- | --- |
| Size | ≤4cm | reference | reference | 0 |
|  | >4cm | 2.37 | 0.8632131 | 58.53383 |
| LVI | Absent | reference | reference | 0 |
|  | Present | 2.45 | 0.8974792 | 60.85739 |
| Silva | Low risk | reference | reference | 0 |
|  | High risk | 4.37 | 1.474725 | 100 |
| depth | ≤1/2 | reference | reference | 0 |
|  | >1/2 | 3.30 | 1.194274 | 80.98285 |

Abbreviations: LVI=lymphovascular invasion; OR=odds ratios.

**Supplementary Table 3 Risk stratification corresponding to the total score**

| Risk | 0.02 | 0.03 | 0.05 | 0.1 | 0.2 | 0.3 | 0.4 | 0.5 | 0.6 |
| --- | --- | --- | --- | --- | --- | --- | --- | --- | --- |
| Total points | 12.63941 | 40.82919 | 76.88071 | 127.54882 | 182.53739 | 219.08634 | 249.04672 | 276.54102 | 304.03532 |

**Supplementary Figure 1 AUC of a model without Silva pattern**
